# Tegoprazan Versus Proton Pump Inhibitors for Erosive Esophagitis: A Meta‐Analysis of Noninferiority Randomized Controlled Trials

**DOI:** 10.1002/jgh3.70298

**Published:** 2025-11-10

**Authors:** Muhammad Imaz Bhatti, Muhammad Safiullah, Azka Ijaz, Qasim Mehmood, Ishmal Fatima Shahid, Zahin Shahriar

**Affiliations:** ^1^ King Edward Medical University Lahore Pakistan; ^2^ Dhaka Medical College Hospital Dhaka Bangladesh

**Keywords:** erosive esophagitis, meta‐analysis, P‐CABs, PPIs, proton pump inhibitors, tegoprazan

## Abstract

**Purpose:**

Proton pump inhibitors (PPIs) remain the standard treatment option for erosive esophagitis (EE) but have notable limitations due to side effects associated with long‐term use. Tegoprazan, a novel potassium‐competitive acid suppressant (P‐CAB), may provide an alternative treatment option. In this study, we aim to evaluate the healing rates of EE using tegoprazan versus PPIs, along with their respective safety profiles.

**Methods:**

A comprehensive literature search was conducted in PubMed, Embase, and ClinicalTrials.gov from inception to March 2025. Risk of bias was evaluated using the Cochrane RoB 2.0 tool. Pooled estimates were calculated using random‐effects models in Review Manager 5.4.

**Results:**

Three RCTs encompassing a total of 766 patients were included in the analysis. Tegoprazan demonstrated comparable healing rates to PPIs at both 4 weeks (risk ratio [RR] 1.05; 95% confidence interval [CI]: 0.99–1.11; *p* = 0.12) and 8 weeks (RR 1.02; 95% CI: 0.98–1.06; *p* = 0.35). Subgroup analyses by dose (50 and 100 mg) showed no significant differences. While overall adverse event rates were similar between groups, the incidence of headache was numerically lower in the tegoprazan group (1.3% vs. 4.2%; RR = 0.40; 95% CI: 0.08–2.00; *p* = 0.26), though this difference was not statistically significant.

**Conclusion:**

Tegoprazan shows comparable efficacy and safety to standard PPIs for treating EE. While healing rates were slightly higher, particularly with the 100 mg dose, the differences were not statistically significant. Tegoprazan may be a suitable alternative for patients intolerant to PPIs, though larger, long‐term, multiethnic studies are warranted to confirm these findings and assess patient‐centered outcomes.

## 1 Introduction

Erosive esophagitis (EE) is a common clinical manifestation of gastroesophageal reflux disease (GERD), arising mainly due to the reflux of acid and pepsin into the esophagus [1]. It affects approximately 30%–40% of the patients with GERD, although a considerable number may remain asymptomatic despite having the condition [2, 3]. Common symptoms of EE include retrosternal chest pain, odynophagia, heartburn, and dysphagia [1]. Patients with EE face an elevated risk of complications such as Barrett's esophagus, esophageal adenocarcinoma, and gastrointestinal bleeding [2]. The prevalence of EE is higher in America, Africa, and Europe compared to Asia, largely attributed to lifestyle factors. The leading risk factors for EE include hiatal hernia, age ≥ 60 years, and obesity [4]. In 2015, the United States spent over $18 billion on managing esophageal disorders [2].

Proton pump inhibitors (PPIs) are the first‐line treatment for EE due to their proven efficacy in mucosal healing and symptom control [5]. However, 57.5% of patients continue to experience heartburn, and 30.1% report regurgitation despite PPI use. Moreover, most patients remain dissatisfied with their treatment and desire more effective, longer‐lasting options, prompting healthcare providers to explore better alternatives to PPIs [6]. Potassium‐competitive acid blockers (P‐CABs) have emerged as a promising therapy for EE due to their rapid, acid‐independent activation. They provide long‐lasting, reversible inhibition of the gastric H^+^/K^+^ ATPase, setting them apart from conventional PPIs [7]. P‐CABs have also demonstrated improved healing outcomes in EE, particularly in more severe cases compared to PPIs [8]. They offer potent and consistent acid suppression, unaffected by patient‐specific factors such as age or genetics, and exhibit stable pharmacokinetics and additional advantages like reduced bleeding risk and ulcer prevention [8, 9, 10].

In this study, we aim to systematically evaluate whether tegoprazan is therapeutically comparable to standard PPIs by pooling and analyzing data from multiple high‐quality noninferiority randomized controlled trials (RCTs).

## 2 Materials and Methods

This meta‐analysis was conducted in accordance with the Cochrane Handbook for Systematic Reviews of Interventions and reported according to the “Preferred Reporting Items for Systematic Reviews and Meta‐Analysis” (PRISMA) statement [11, 12]. This study's protocol was registered in the International Prospective Register of Systematic Reviews (PROSPERO) with the registration number CRD420251030505. Our study did not require ethical approval.

### 2.1 Data Sources and Search Strategy

A comprehensive literature search was conducted to identify relevant studies on Tegoprazan for EE. We searched PubMed, Embase (via Ovid), and ClinicalTrials.gov from inception to March 2025. No language restrictions were applied. Additionally, the reference lists of included articles and relevant systematic reviews were manually screened to identify any further eligible studies. A detailed search strategy using keywords and Medical Subject Headings (MeSH) terms related to Tegoprazan, EE, and PPIs is provided in Table S1.

### 2.2 Eligibility Criteria

Studies were included if they met the following criteria: (i) enrolled adult patients with endoscopically confirmed EE (Population); (ii) evaluated tegoprazan as the intervention; (iii) used any PPI, such as esomeprazole or lansoprazole, as the comparator; (iv) reported at least one relevant clinical outcome, including endoscopic healing rates, or adverse events; and (v) utilized a RCT design.

Studies were excluded if they: (i) were not published in English; (ii) were reviews, letters, case series, conference proceedings, or unpublished studies; or (v) did not provide sufficient or usable data for analysis.

### 2.3 Study Selection and Data Extraction

All identified records were first imported to Mendeley Reference Manager (version 2.122.1; Elsevier Ltd., Amsterdam, Netherlands) [13] and duplicate articles were removed. Two reviewers (M.I.B. and A.I.) independently examined the titles and abstracts. Full‐text articles were then assessed against the inclusion criteria. Any disagreements were resolved through discussion with a third, independent author (M.S.).

After the study selection, data were extracted into a predefined Excel spreadsheet by two authors (M.I.B. and A.I.) to corroborate the consistency of the studies. Any disagreement was resolved by discussion with a third author (M.S.). The following data items were extracted from each included study: study ID, year of publication, country, study design, underlying condition (EE), experimental intervention and its route of administration, comparator intervention, total number of patients, gender distribution, mean age, *Helicobacter pylori* infection status, baseline severity of EE, alcohol consumption, smoking status, follow‐up duration, and reported clinical outcomes (endoscopic healing rates, and adverse events).

### 2.4 Bias Assessment

The risk‐of‐bias assessment was conducted using the revised Cochrane Risk of Bias tool for randomized trials (RoB 2.0), outlined in the Cochrane Handbook [14]. Each study was independently evaluated across multiple domains, including the randomization process, deviations from intended interventions, missing outcome data, outcome measurement, and selection of reported results. The risk of bias was classified as low, high, or raising some concerns.

### 2.5 Data Synthesis

Statistical analysis was conducted using Review Manager (RevMan, version 5.4; The Cochrane Collaboration, Copenhagen, Denmark). Dichotomous outcomes were expressed as relative risk (RR) with 95% confidence intervals (CIs). Meta‐analyses were performed using a random‐effects model with the Mantel–Haenszel method. A two‐tailed *p* < 0.05 was considered statistically significant. The *I* ^2^ index and chi‐square test were applied to assess heterogeneity, with a *p* < 0.1 indicating significant heterogeneity among the included studies. To avoid the unit‐of‐analysis error arising from the three‐armed trial by Lee et al. [15], in which the esomeprazole 40 mg group served as a common comparator for both tegoprazan doses, we split the esomeprazole group into two equal halves. Each half was treated as an independent comparator for one tegoprazan dose group, as recommended by the Cochrane Handbook (section 23.3.4) [11].

We also conducted a subgroup analysis to assess healing outcomes at two time points: 4 and 8 weeks. Moreover, a subgroup analysis was done to compare the effects of two dosage groups (50 vs. 100 mg). These analyses were performed to evaluate time‐dependent and dose‐dependent variations in healing outcomes. To avoid inappropriate pooling of different tegoprazan doses (50 and 100 mg) and PPI types (esomeprazole 40 mg and lansoprazole 30 mg), we also conducted a network meta‐analysis (NMA) using a frequentist random‐effects model. The analysis was performed in R (version 4.4.2). Network plots were constructed to visualize the connections between treatment nodes for each outcome (Figure S1).

Because all included studies were designed as noninferiority RCTs, each with its own prespecified noninferiority margin, the current meta‐analysis synthesized results from these trials to assess comparative efficacy and safety. No new or independent noninferiority margin was applied at the meta‐analysis level.

Publication bias was not assessed due to a smaller number of studies. Publication bias assessment is not recommended when the number of included studies is small, typically fewer than 10. In such cases, statistical tests for funnel plot asymmetry, such as Egger's test or Begg's test, lack the power to detect true bias and may produce misleading results. These tests are more reliable when applied to meta‐analyses with a larger number of studies, where the detection of asymmetry is more robust and meaningful [16].

### 2.6 Certainty of Evidence Assessment

To assess the certainty of evidence for each outcome, we applied the five GRADE (Grading of Recommendations, Assessment, Development, and Evaluation) criteria. These include evaluating study limitations, consistency of results, imprecision, indirectness, and potential publication bias. The summary of findings (SoF) table was generated using GRADEPro GDT [17, 18].

## 3 Results

### 3.1 Study Selection and Characteristics of Included Studies

**FIGURE 1:**
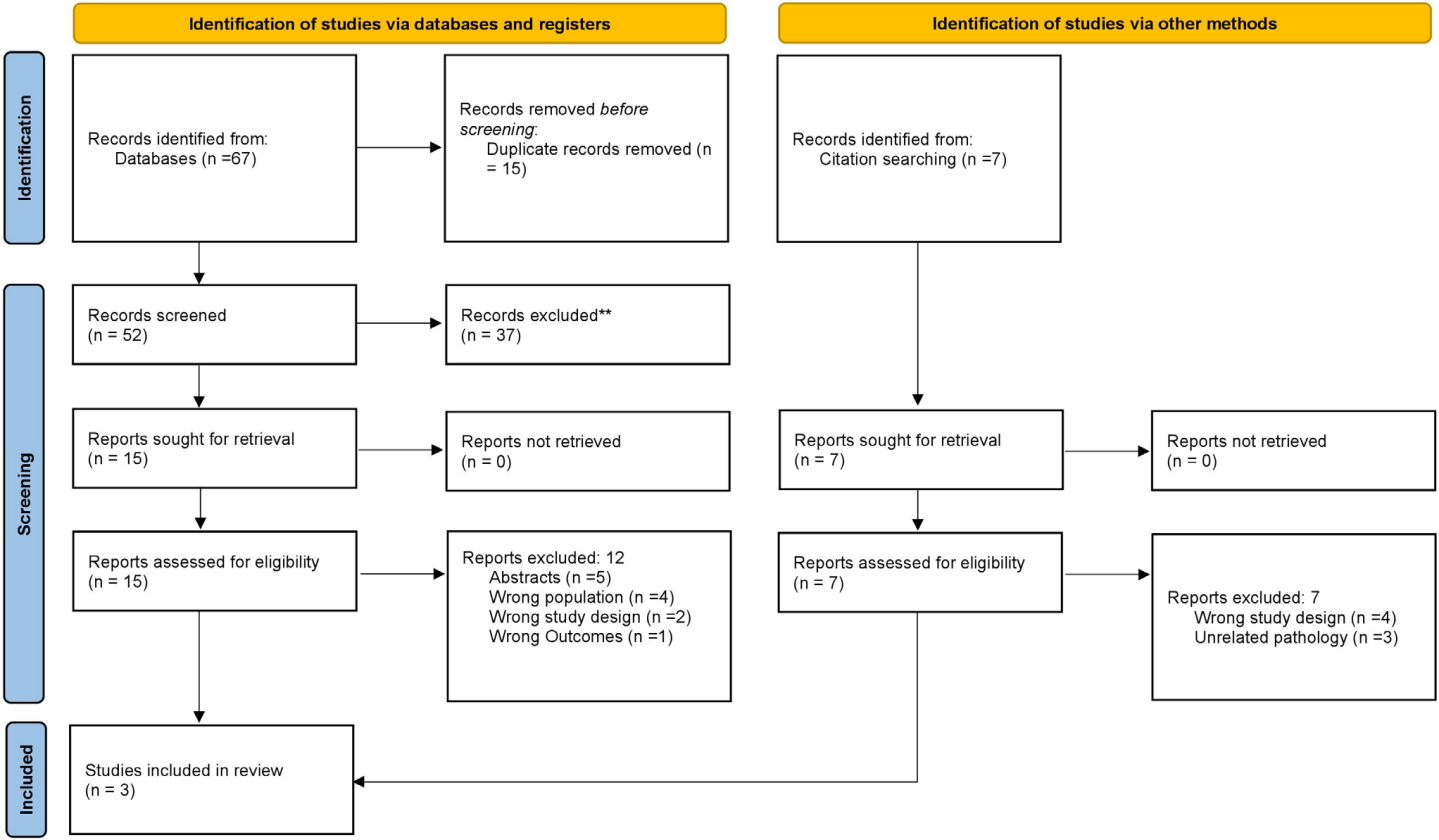
PRISMA flowchart.

An initial database search identified 67 potential studies, with an additional seven records found through citation searching. After removing duplicates and screening studies based on their titles and abstracts, we assessed 15 full‐text manuscripts for eligibility. Subsequently, three RCTs were selected to be included in our study. The detailed study selection process is illustrated in the PRISMA flowchart (Figure 1).

Three RCTs [9, 15, 19] compared the efficacy of tegoprazan versus PPIs in patients with EE. The included studies enrolled a total of 766 patients with endoscopically confirmed EE (LA grades A–D). All trials used tegoprazan 50 mg daily, with one also evaluating 100 mg [15]. Comparators were esomeprazole 40 mg (two trials) or lansoprazole 30 mg (one trial). Treatment duration ranged from 4 to 8 weeks. Patients were predominantly male (55%–73%) with a mean age of 46.9–53 years. *H. pylori* positivity ranged from 7% to 12.5% (reported in two studies). Most had LA grade A/B disease (67%–85%), with alcohol use (14%–40%) and smoking (5%–21%) commonly reported. Table 1 summarizes the demographics and baseline characteristics of all included studies.

**TABLE 1:** Demographic and baseline characteristics of included studies.

Study ID		Lee et al. [15]	Zhu et al. [19]	Shin et al. [9]
Country		South Korea	China	South Korea
Study design		RCT (randomized, double‐blinded, multicentric)	RCT (randomized, double‐blinded, multicentric)	RCT (randomized, double‐blinded, multicentric)
Condition included		Erosive esophagitis (LA Grades A–D)	Erosive esophagitis (LA Grades A–D)	Erosive esophagitis (LA Grades A–D)
Experimental intervention		Tegoprazan 50 mg or 100 mg qd for 4–8 weeks (oral)	Tegoprazan 50 mg qd for 4–8 weeks (oral)	Tegoprazan 50 mg qd for 2–4 weeks (oral)
Comparator intervention		Esomeprazole 40 mg qd for 4–8 weeks	Esomeprazole 40 mg qd + placebo of tegoprazan for 4–8 weeks	Lansoprazole 30 mg qd for 2–4 weeks
Number of patients		300 (99 vs. 102 vs. 99)^a^	248 (123 vs. 125)	218 (109 vs. 109)
Male, *n* (%)		181 (60%)	182 (73%)	136 (62%)
Mean age, years (mean ± SD)		51.97 ± 10.55	46.9 ± 11.55	53.0 ± 15.5
*H. pylori* positive, *n*		23 vs. 21 vs. 21^a^	31 vs. 30	Excluded (per protocol)
Baseline erosive esophagitis, *n* (%)	LA Grade A	199 (67%)	112 (45%)	129 (59.2%)
	LA Grade B	88 (29%)	115 (46%)	70 (32.1%)
	LA grade C/D	13 (4%)	21 (8%)	19 (8.7%)
Alcohol consumption, *n*		42 vs. 45 vs. 39^a^	30 vs. 30	40 vs. 43
Smoking, *n*		27 vs. 17 vs. 15^a^	47 vs. 43	17 vs. 21
Follow‐up duration		8 weeks	8 weeks	4 weeks

Abbreviations: LA Grades A–D, Los Angeles Classification Grades A–D; PPI, proton pump inhibitor; qd, once daily; RCT, randomized controlled trial.

^a^ Tegoprazan 50 mg vs. Tegoprazan 100 mg vs. PPI.

### 3.2 Risk of Bias Assessment

**FIGURE 2:**
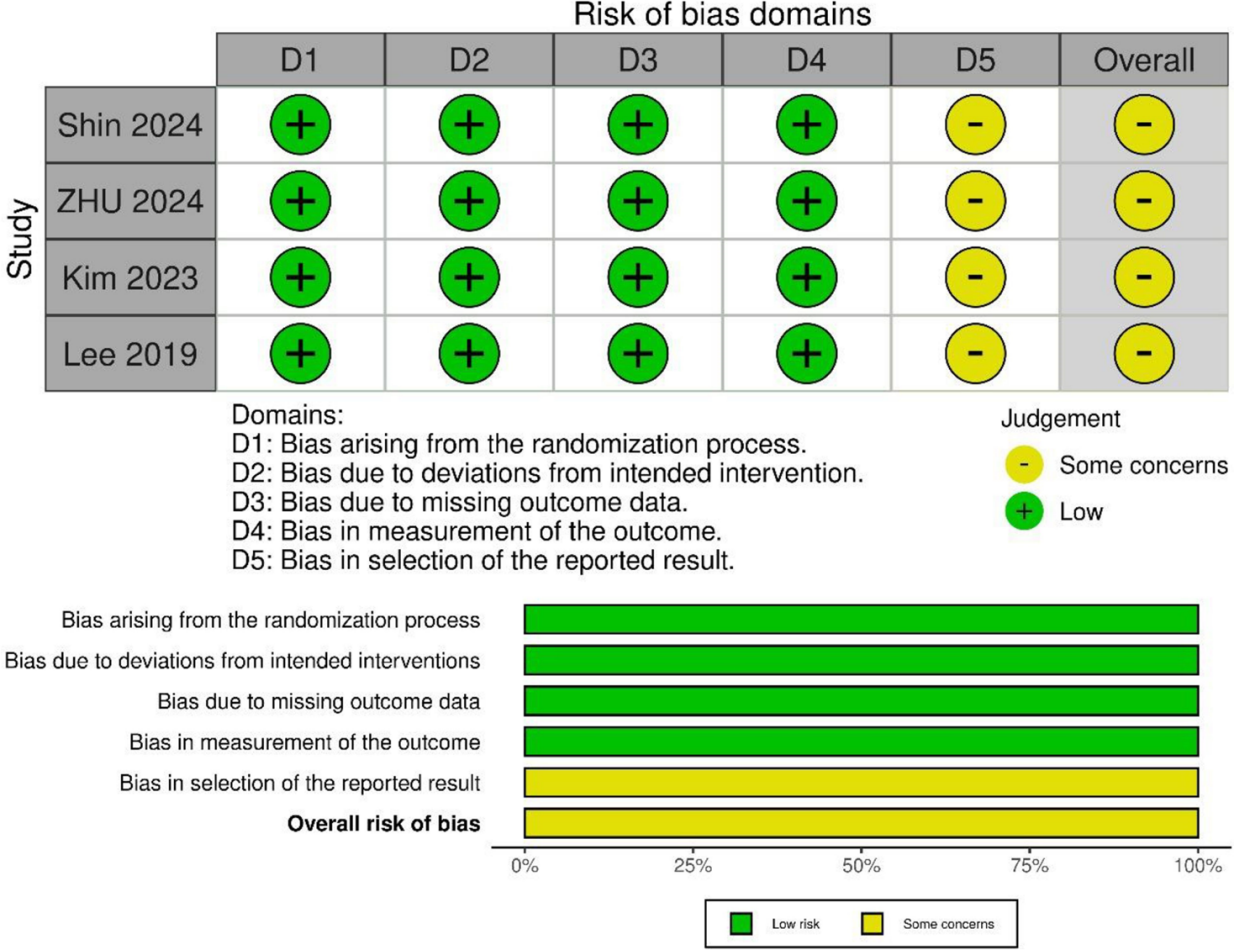
Summary of risk of bias assessment.

All three included RCTs [9, 15, 19] were evaluated using the Cochrane RoB 2.0 tool. Across domains D1 (bias arising from the randomization process), D2 (bias due to deviations from intended interventions), D3 (bias due to missing outcome data), and D4 (bias in measurement of the outcome), all studies demonstrated a low risk of bias. However, each study was rated as having some concerns in D5 (bias in selection of the reported result), due to a lack of access to prespecified analysis protocols. Consequently, the overall risk of bias was judged as “some concerns” for all studies (Figure 2).

### 3.3 Results of Synthesis

#### 3.3.1 Endoscopic Healing Rates at 4‐ and 8‐Week

**FIGURE 3:**
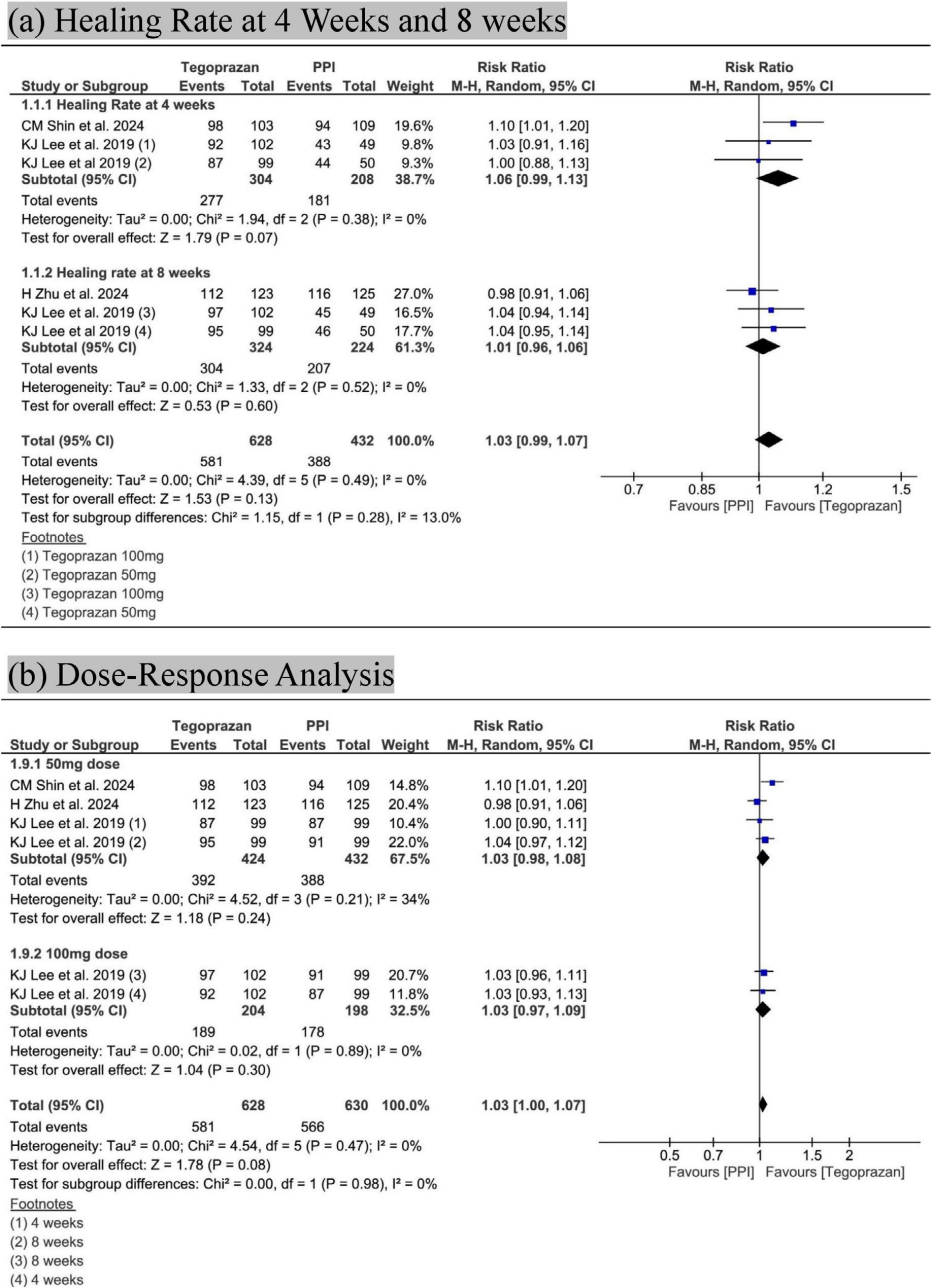
Forest plots showing: (a) comparative healing rates between Tegoprazan and proton pump inhibitors (PPIs) at 4 and 8 weeks and (b) subgroup analysis comparing healing rates between Tegoprazan 50 mg, Tegoprazan 100 mg, and PPIs.

At 4 weeks, tegoprazan showed a 91.1% healing rate compared to 87% for PPIs, with no statistically significant difference (RR 1.06; 95% CI: 0.99–1.13; *p* = 0.07; *I* ^2^ = 0%) (Figure 3a). The quality of evidence was rated as high according to GRADE (Table 2).

**TABLE 2:** Grading recommendations assessment, development, and evaluation (GRADE) summary of findings.

Outcome		No. of studies	Anticipated absolute effects (95% CI)	Risk of bias	Inconsistency	Indirectness	Imprecision	Publication bias	Quality of evidence (GRADE)
Healing rate	**At 4 weeks**	2 RCTs	**52 more per 1000** (from 9 fewer to 113 more)	Not serious	Not serious	Not serious	Not serious	Undetected	⨁⨁⨁⨁ High
	**At 8 weeks**	2 RCTs	**9 more per 1000** (from 37 fewer to 55 more)	Not serious	Not serious	Not serious	Not serious	Undetected	⨁⨁⨁⨁ High
Dose response	50 mg	3 RCTs	**27 more per 1000** (from 18 fewer to 72 more)	Not serious	Not serious	Not serious	Not serious	Undetected	⨁⨁⨁⨁ High
Treatment‐emergent adverse events (TEAEs)		3 RCTs	**26 more per 1000** (from 46 fewer to 115 more)	Not serious	Not serious	Not serious	Very serious	Undetected	⊕ ⊕ ⊝⊝ Low
Any Gastrointestinal disorder		2 RCTs	**6 fewer per 1000** (from 32 fewer to 45 more)	Not serious	Not serious	Not serious	Very serious	Likely	⊕⊝⊝⊝ Very low
Headache		2 RCTs	**20 fewer per 1000** (from 31 fewer to 38 more)	Not serious	Serious	Not serious	Very serious	Undetected	⊕⊝⊝⊝ Very low
Erosive gastritis		2 RCTs	**2 fewer per 1000** (from 24 fewer to 52 more)	Not serious	Not serious	Not serious	Very serious	Likely	⊕⊝⊝⊝ Very low

*Note:* Bolded values shows significant values (*p* < 0.05).

At 8 weeks, the healing rates were 93.8% for tegoprazan and 92.4% for PPIs, again showing no significant difference (RR 1.01; 95% CI: 0.96–1.06; *p* = 0.60; *I* ^2^ = 0%) (Figure 3a). GRADE classified the evidence as high quality (Table 2).

In the pooled analysis of both time points, tegoprazan demonstrated a slightly higher overall healing rate (92.5%) than PPIs (89.8%), though the difference remained nonsignificant (RR 1.03; 95% CI: 0.99–1.07; *p* = 0.13; *I* ^2^ = 0%), No significant subgroup difference was observed between the 4‐ and 8‐week analyses (Figure 3a).

#### 3.3.2 Dose–Response Analysis

For the 50 mg dose, tegoprazan showed a healing rate of 92.4% versus 89.8% in the PPI group (RR 1.03; 95% CI: 0.98–1.08; *p* = 0.24; *I* ^2^ = 34%) (Figure 3b). The quality of evidence, as determined by GRADE, was ranked to be high (Table 2). The 100 mg dose resulted in a healing rate of 92.6% compared to 89.9% with PPIs (RR 1.03; 95% CI: 0.97–1.09; *p* = 0.30; *I* ^2^ = 0%) (Figure 3b). No significant subgroup difference was observed between the two doses (*p* = 0.98).

### 3.4 Adverse Events

#### 3.4.1 Treatment‐Emergent Adverse Events (TEAEs)

**FIGURE 4:**
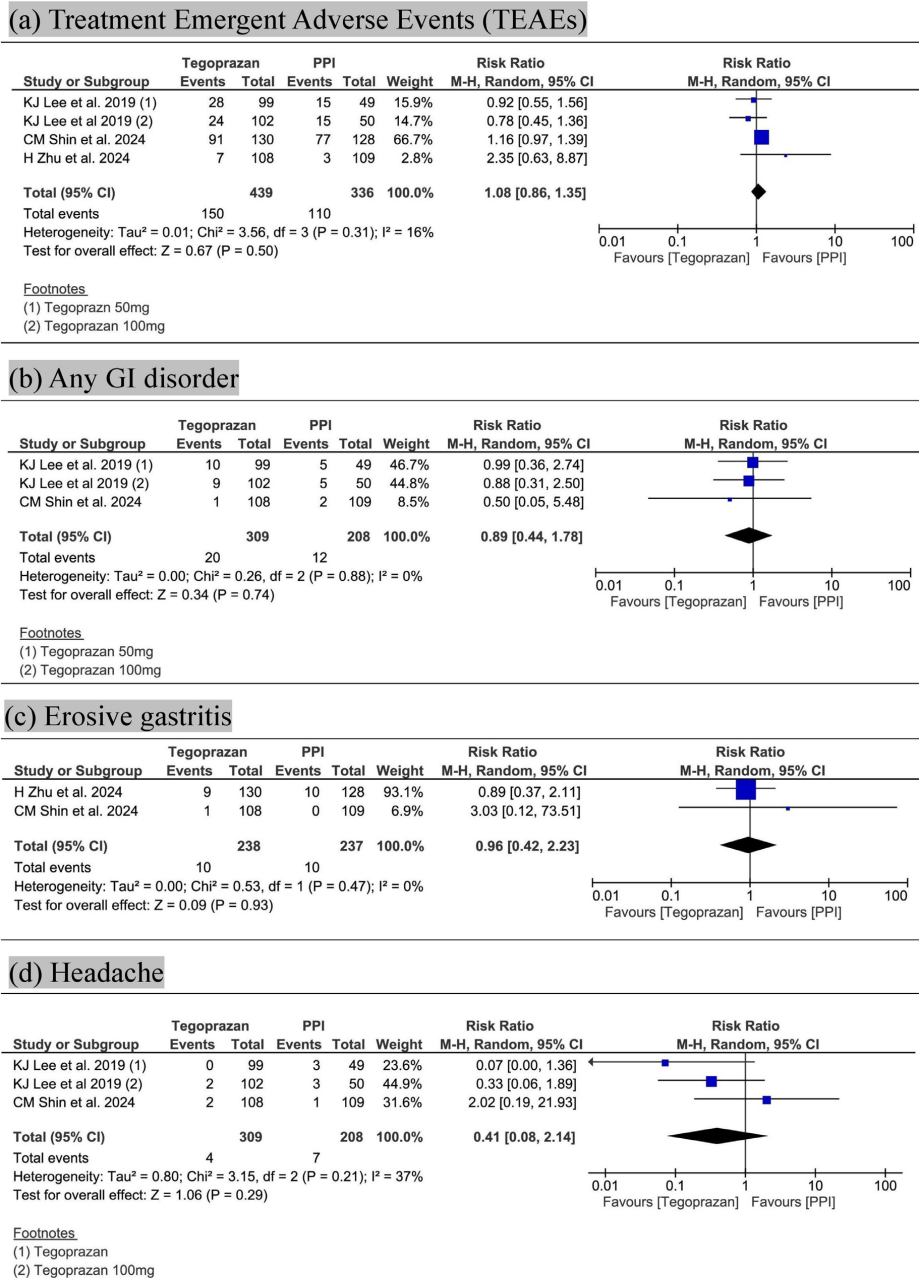
Forest plots comparing Tegoprazan and proton pump inhibitors (PPIs) for: (a) treatment‐emergent adverse events (TEAEs); (b) gastrointestinal (GI) disorders; (c) erosive gastritis; and (d) headache.

TEAEs occurred in 34.2% of tegoprazan‐treated patients and 32.7% of PPI‐treated patients (RR 1.08; 95% CI: 0.86–1.35; *p* = 0.50; *I* ^2^ = 16%) (Figure 4). Based on GRADE, the quality of evidence was low, primarily due to imprecision (Table 2).

#### 3.4.2 Gastrointestinal Disorders

Gastrointestinal events occurred in 6.5% of patients receiving tegoprazan and 5.7% receiving PPIs (RR 0.89; 95% CI: 0.44–1.78; *p* = 0.74; *I* ^2^ = 0%) (Figure 4). The GRADE assessment rated the certainty of evidence as very low, due to imprecision (Table 2).

#### 3.4.3 Erosive Gastritis

Erosive gastritis incidence was identical in both groups (4.2% each; RR 0.96; 95% CI: 0.42–2.23; *p* = 0.93; *I* ^2^ = 0%) (Figure 4). GRADE rated the evidence as very low owing to imprecision (Table 2).

#### 3.4.4 Headache

Headache was less frequent with tegoprazan (1.3%) compared to PPIs (3.4%), though the difference was not statistically significant (RR 0.41; 95% CI: 0.08–2.14; *p* = 0.29; *I* ^2^ = 37%) (Figure 4). The quality of evidence was very low, due to imprecision and inconsistency (Table 2).

#### 3.4.5 NMA

Lansoprazole 30 mg demonstrated significantly lower healing rates compared to both tegoprazan 50 mg (RR 0.91; 95% CI: 0.83–0.99) and tegoprazan 100 mg (RR 0.89; 95% CI: 0.81–0.99), while no significant differences were observed between esomeprazole 40 mg and either dose of tegoprazan (Table S2 and Figure S2). For safety outcomes including TEAEs, gastrointestinal disorders, erosive gastritis, and headache, there were no statistically significant differences among the compared interventions.

## 4 Discussion

This meta‐analysis demonstrates that tegoprazan is comparable to conventional PPIs in achieving endoscopic healing in patients with EE, with a favorable safety profile. Healing rates were high across both 4‐ and 8‐week treatment periods, with no significant differences in efficacy or safety outcomes between tegoprazan and PPIs. Additionally, subgroup analyses based on treatment duration and dosage revealed consistent results, supporting the robustness and sustained efficacy of tegoprazan. These findings reinforce the therapeutic value of tegoprazan as a viable alternative to PPIs in the management of EE.

The comparable efficacy of tegoprazan and PPIs can be explained by their shared objective of reducing gastric acid secretion, achieved via different mechanisms. Tegoprazan is a potassium‐competitive acid blocker (P‐CAB) that binds reversibly to the gastric H^+^/K^+^‐ATPase, thereby providing rapid and consistent acid suppression independent of proton pump activation or meal timing [20]. In contrast, PPIs require activation in an acidic environment, leading to a delayed onset of action and variability influenced by food intake and metabolic factors [21]. This results in faster onset of action and a more predictable pharmacodynamic profile using P‐CABs, which may translate to earlier symptom relief and enhanced mucosal protection, even if the healing rates remain numerically similar [22]. This is in comparison to a study conducted by Wang et al. [23], which showed that there are no significant differences in the healing rates when comparing tegoprazan with other P‐CABs and traditional PPIs. Another study by Abdel‐Aziz et al. [24] concluded that despite having some potential advantages over PPIs, tegoprazan demonstrates comparable efficacy for healing peptic ulcers and other acid reflux diseases.

Importantly, the clinical significance of these comparable findings extends beyond statistical equivalence. For patients with suboptimal or partial response to PPIs, or those with rapid symptom recurrence between doses, tegoprazan's consistent acid suppression may reduce nighttime symptoms and improve quality of life, particularly for patients with moderate‐to‐severe EE or nocturnal reflux [9, 25]. Moreover, tegoprazan may improve compliance due to once‐daily dosing, which is not contingent upon food intake, unlike many PPIs that must be taken before eating to be fully effective [26].

From a pharmacogenomic perspective, tegoprazan's independence from CYP2C19 metabolism offers another advantage, especially in populations with a high prevalence of CYP2C19 polymorphisms, such as East Asians [25, 27]. Poor metabolizers of PPIs often experience reduced drug clearance, leading to variability in acid suppression and symptom control [28]. Tegoprazan, metabolized via different pathways, avoids this issue, providing more consistent efficacy across different genotypes [25]. However, this also underscores the need for studies in populations with different CYP2C19 allele frequencies (e.g., Caucasians, Africans), as the clinical benefit of tegoprazan may vary by ethnicity.

The subgroup analysis in this study is particularly valuable, as it shows that tegoprazan's efficacy is consistent across treatment durations and doses, suggesting that its therapeutic effects are not transient but durable over time. Furthermore, the data suggest that even the lower 50 mg dose of tegoprazan may be clinically sufficient in most patients, which could reduce drug exposure, costs, and potential side effects [9]. Given its once‐daily dosing regimen, tegoprazan may also improve patient compliance, though this should be formally evaluated in future trials through objective adherence measures [15].

In evaluating safety and tolerability, our analysis found a slightly higher incidence of TEAEs in the tegoprazan group compared to the PPI group; however, this difference was not statistically significant. Interestingly, the frequency of headaches was lower among patients treated with tegoprazan, though again without reaching statistical significance. These findings are consistent with the study by Wang et al. [23], which compared the overall adverse event profiles of tegoprazan and conventional PPIs in patients with EE and similarly reported no significant differences in the safety outcomes between the treatment arms.

From a real‐world standpoint, barriers to widespread adoption of tegoprazan include limited availability in many countries, particularly in low‐ and middle‐income nations. Currently, tegoprazan is approved mainly in South Korea and a few other Asian countries, and has not yet been integrated into major global clinical guidelines [29, 30]. High development costs, lack of generic availability, and regulatory delays contribute to its limited accessibility [31, 32]. Additionally, physicians may be hesitant to switch from PPIs due to the long‐standing familiarity, lower cost, and inclusion of PPIs in most current guideline algorithms for GERD and EE [30]. Nonetheless, as more head‐to‐head data become available and real‐world effectiveness is demonstrated, tegoprazan could be considered an alternative or adjunct to PPIs in select populations.

### 4.1 Clinical and Research Implications

Future research should focus on conducting large‐scale, multinational, prospective RCTs that include populations from both high‐income and low‐ to middle‐income countries to better assess the real‐world effectiveness of tegoprazan. These studies should incorporate diverse genetic and ethnic backgrounds, particularly given the varying prevalence of CYP2C19 polymorphisms and dietary factors that may influence gastric acid production. Extended follow‐up periods will be important for evaluating the long‐term durability of mucosal healing, recurrence rates, and safety outcomes. Additional investigation is also needed to assess whether tegoprazan may be used in combination with PPIs in refractory cases or serve as a second‐line agent in patients with incomplete symptom control. While such approaches are not currently reflected in clinical guidelines, they represent areas for potential exploration. Future trials should aim for treatment adherence monitoring and evaluate cost‐effectiveness as well as patient‐reported outcomes. Finally, head‐to‐head comparisons with other P‐CABs, newer PPI formulations, and combination strategies could help define tegoprazan's place within the broader treatment landscape for EE.

### 4.2 Limitations

This study, despite concluding the comparable efficacy and safety of tegoprazan over conventional PPIs, has certain limitations. Firstly, the sample size was limited as we only included RCTs comparing the efficacy and safety of tegoprazan versus traditional PPIs. Secondly, the included studies were mainly conducted in Asian countries, like China and Korea, which raises concerns about the efficacy of this drug among people residing in other demographic locations. We also acknowledge some variability in treatment durations and dosing regimens across studies. However, subgroup analyses did not reveal significant heterogeneity in efficacy outcomes based on treatment length or dosage. Nevertheless, standardization in future studies would enhance interpretability and enable more precise comparisons. In addition, subgroup analysis based on grades of healing was not possible, given the limited number of available studies.

The risk of bias assessment revealed some concerns, emphasizing the need for more rigorously designed and adequately powered clinical trials. The certainty of evidence, assessed using GRADE, ranged from very low to high. Lower ratings were largely attributed to imprecision, inconsistency, or both, particularly in safety outcomes. Publication bias was not formally evaluated due to the small number of included studies (< 10), which limits the interpretability of funnel plots and associated tests. However, the possibility of publication bias cannot be excluded and remains a potential limitation.

Furthermore, although statistical heterogeneity was generally low, we acknowledge that this does not preclude the presence of potential clinical heterogeneity, particularly related to differences in dosing regimens and comparator PPIs. This aspect should be interpreted with caution and addressed in future head‐to‐head or NMA to strengthen the robustness of comparative evidence.

## 5 Conclusion

Our meta‐analysis concludes that tegoprazan demonstrates comparable efficacy and safety to PPIs in healing EE. These findings support tegoprazan as a viable alternative, particularly for patients with suboptimal responses to PPIs or CYP2C19 polymorphisms. Broader access, cost‐effectiveness analyses, and rigorous long‐term data in diverse populations are essential to determine its role in future clinical practice.

## Disclosure

No previously published copyrighted material has been reproduced in this manuscript.

## Ethics Statement

As this is a meta‐analysis of previously published studies, ethical approval was not required.

## Consent

This study involved secondary analysis of published data and did not involve direct interaction with human participants.

## Conflicts of Interest

The authors declare no conflicts of interest.

## Supporting information


**Table S1:** Detailed database search strategy.
**Figure S1:** Network plots for (a) healing rate and TEAEs, (b) GI disorders and headache, and (c) erosive gastritis.
**Table S2:** Network meta‐analysis league table.
**Figure S2:** Forest plots.

## Data Availability

All data generated or analyzed during this study are included in this published article [and its supplementary information files, if applicable]. Further inquiries can be directed to the corresponding author.
