# Supplementary material for: Tegoprazan Versus Proton Pump Inhibitors for Erosive Esophagitis: A Meta‐Analysis of Noninferiority Randomized Controlled Trials
Source: JGH Open. 2025 Nov 10;9(11):e70298. doi: 10.1002/jgh3.70298 (PMC12602160; doi:10.1002/jgh3.70298)

**Supplementary Material**

**Tegoprazan versus Proton Pump Inhibitors for Erosive Esophagitis: A Meta-Analysis of Non-Inferiority Randomized Controlled Trials**

**Supplementary Table S1:** Detailed Database Search Strategy

| **Database** | **Search String** |
| --- | --- |
| PubMed, MEDLINE  (15 Results) | (((Tegoprazan) OR (LXI-15028)) OR (CJ-12420)) AND (("Esophagitis"[Mesh]) OR (Esophagitides)) |
| Embase  (50 results) | ('tegoprazan'/exp OR 'tegoprazan' OR 'lxi 15028' OR 'cj 12420') AND ('esophagitis'/exp OR 'esophagitis' OR esophagitides) |
| ClinicalTrials.gov  (2 results) | Tegoprazan AND erosive esophagitis |

**
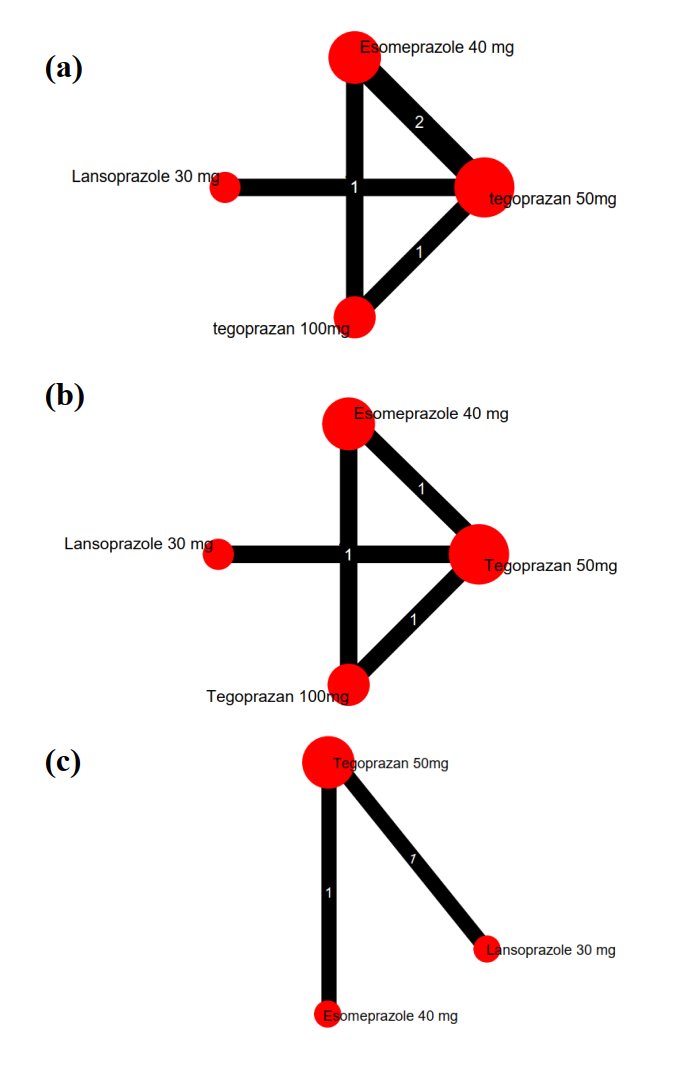
**

**Supplementary Figure S1:** Network plots for **(a)** Healing rate and TEAEs **(b)** GI disorders and headache **(c)** Erosive Gastritis

**Supplementary Table S2:** Network meta-analysis league table

| **Healing rate** | | | |
| --- | --- | --- | --- |
| Esomeprazole 40 mg | . | 0.97 (0.91 to 1.03) | 0.99 (0.95 to 1.04) |
| 1.10 (0.99 to 1.21) | Lansoprazole 30 mg | . | 0.91 (0.83 to 0.99) |
| 0.98 (0.93 to 1.04) | **0.89 (0.81 to 0.99)** | Tegoprazan 100mg | 1.01 (0.95 to 1.07) |
| 0.99 (0.95 to 1.04) | **0.91 (0.83 to 0.99)** | 1.02 (0.96 to 1.07) | Tegoprazan 50mg |
| **TEAEs** | | | |
| Esomeprazole 40 mg | . | 1.29 (0.81 to 2.04) | 0.98 (0.65 to 1.48) |
| 1.14 (0.73 to 1.79) | Lansoprazole 30 mg | . | 0.86 (0.72 to 1.03) |
| 1.23 (0.78 to 1.95) | 1.08 (0.66 to 1.78) | Tegoprazan 100mg | 0.83 (0.52 to 1.33) |
| 0.98 (0.65 to 1.48) | 0.86 (0.72 to 1.03) | 0.79 (0.50 to 1.26) | Tegoprazan 50mg |
| **Any GI disorder** | | | |
| Esomeprazole 40 mg | . | 1.14 (0.49 to 2.70) | 1.00 (0.44 to 2.30) |
| 0.50 (0.04 to 6.31) | Lansoprazole 30 mg | . | 1.98 (0.18 to 21.53) |
| 1.14 (0.49 to 2.70) | 2.27 (0.18 to 28.62) | Tegoprazan 100mg | 0.87 (0.37 to 2.06) |
| 1.00 (0.44 to 2.30) | 1.98 (0.18 to 21.53) | 0.87 (0.37 to 2.06) | Tegoprazan 50mg |
| **Erosive Gastritis** | | | |
| Esomeprazole 40 mg | . | 1.13 (0.47 to 2.69) | Tegoprazan 100mg N/A for outcome |
| 3.42 (0.13 to 93.12) | Lansoprazole 30 mg | 0.33 (0.01 to 8.02) |  |
| 1.13 (0.47 to 2.69) | 0.33 (0.01 to 8.02) | Tegoprazan 50mg |  |
| **Headache** | | | |
| Esomeprazole 40 mg | . | 2.68 (0.64 to 11.22) | 13.00 (0.74 to 227.68) |
| 26.24 (0.63 to 1090.06) | Lansoprazole 30 mg | . | 0.50 (0.05 to 5.38) |
| 2.68 (0.64 to 11.22) | 0.10 (0.00 to 4.80) | Tegoprazan 100mg | 4.85 (0.24 to 99.84) |
| 13.00 (0.74 to 227.68) | 0.50 (0.05 to 5.38) | 4.85 (0.24 to 99.84) | Tegoprazan 50mg |

***Bold*** *indicates significant value*

**Supplementary Figure S2:** Forest plots


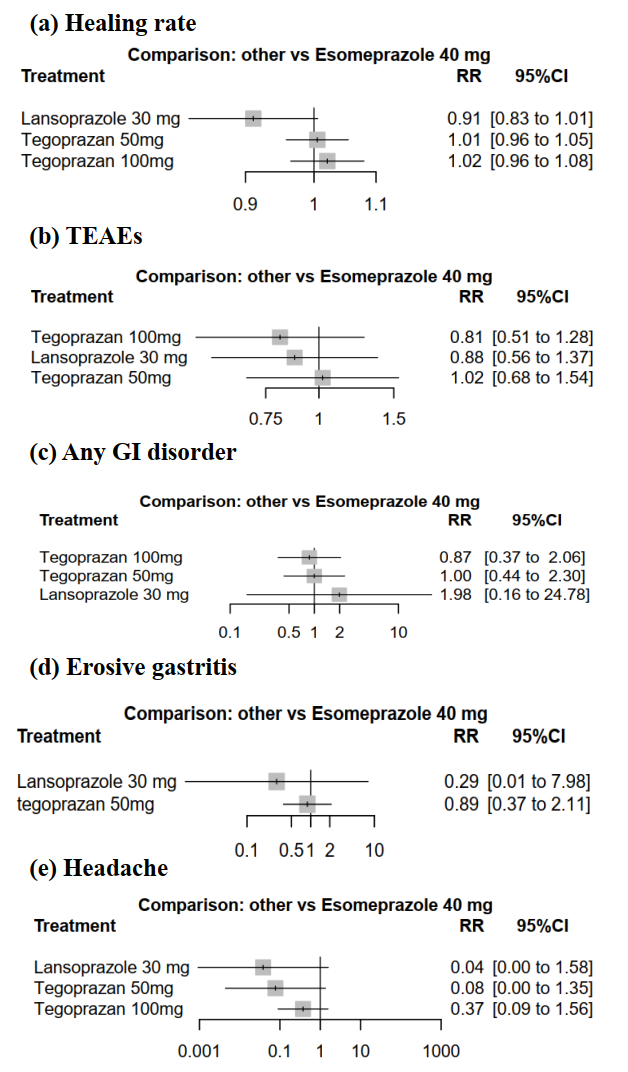

Supplement: Supplementary file 1 — Table S1: Detailed database search strategy. Figure S1: Network plots for (a) healing rate and TEAEs, (b) GI disorders and headache, and (c) erosive gastritis. Table S2: Network meta‐analysis league table. Figure S2: Forest plots. [file JGH3-9-e70298-s001.docx]
